# The Influence of Surgical Realignment Procedures on Dynamic Patellar Tracking: A Dynamic Magnetic Resonance Imaging-Controlled Feasibility Study

**DOI:** 10.3390/diagnostics12112761

**Published:** 2022-11-11

**Authors:** Jannik Frings, Tobias Dust, Jennifer Meyer, Matthias Krause, Karl-Heinz Frosch, Gerhard Adam, Frank Oliver Henes, Clemens Spink, Kai-Jonathan Maas

**Affiliations:** 1 Department of Trauma and Orthopaedic Surgery, University Medical Center Hamburg-Eppendorf, 20251 Hamburg, Germany; 2 Department of Trauma Surgery, Orthopaedics and Sports Traumatology, BG Hospital Hamburg, 21033 Hamburg, Germany; 3 Department of Diagnostic and Interventional Radiology and Nuclear Medicine, University Medical Center Hamburg-Eppendorf, 20251 Hamburg, Germany; 4 Department of Diagnostic and Interventional Radiology, BG Hospital Hamburg, 21033 Hamburg, Germany

**Keywords:** patella, instability, maltracking, realignment procedure, dynamic MRI

## Abstract

Persisting patellar maltracking following surgical realignment often remains unseen. The aim of this study was to analyze the effects of realignment procedures on patellofemoral kinematics in patients with patellofemoral instability (PFI) and patellofemoral maltracking (PM) by using dynamic magnetic resonance imaging (MRI). Patients planned for surgical patellar realignment due to PFI and a clinically and radiologically apparent PM between December 2019 and May 2022 were included. Patients without PM, limited range of motion, joint effusion, or concomitant injuries were excluded. Dynamic mediolateral translation (dMPT) and patella tilt (dPT) were measured preoperatively and three months postoperatively. In 24 patients (7 men, 17 women; mean age 23.0 years), 10 tibial tubercle transfers, 5 soft tissue patella tendon transfers, 6 trochleoplasties, 3 lateral lengthenings, 1 varizating distal femoral osteotomy (DFO), and 1 torsional DFO were performed. At final follow-up, dMPT (from 10.95 ± 5.93 mm to 4.89 ± 0.40 mm, *p* < 0.001) and dPT (from 14.50° ± 10.33° to 8.44° ± 7.46°, *p* = 0.026) were significantly improved. All static radiological parameters were corrected to physiological values. Surgical patellar realignment contributed to the significant improvement of patellofemoral kinematics, with an approximation to normal values. The postoperative application of dynamic MRI allowed for a quantification of the performed correction, allowing for a postoperative control of success.

## 1. Introduction

Patellofemoral instabilities are often associated with concomitant anatomical pathologies, which contribute to an increased risk of recurring patellar dislocations [1,2,3]. Specific risk stratification models have been developed to predict the subsequent risk of recurrence under nonoperative treatment following primary patellar dislocation [1,3,4]. Among the risk factors, those associated with lateral patellar maltracking have been found to be the most relevant [5]. This observation was confirmed by multiple biomechanical investigations that demonstrated direct alterations of patellar tracking when certain risk factors were present [6,7,8,9,10]. On the basis of these findings, modern surgical approaches are aimed at identifying and addressing all relevant pathologies in the affected knee [11,12,13]. In the recent literature, several clinical studies have examined the postoperative success of individualized patellofemoral surgery, and most of these studies reported improved patient-reported outcome measures (PROMs) and low redislocation rates [14,15,16,17,18,19,20,21]. However, successful correction of the causative patellar maltracking remains unverifiable, as neither static magnetic resonance (MR) imaging (MRI) nor clinical examination can provide sufficient objective information [22]. With the implementation of dynamic MRI sequences, patellofemoral diagnostics have been supplemented with direct visualization, and thus patellar maltracking measurement [23,24,25]. While originally described to enhance preoperative diagnostics, dynamic MRI may also serve its purpose in the postoperative course. This is the first study to show that verification of a successfully corrected patellar maltracking may be possible.

The purpose of this study was to test the postoperative application of dynamic MRI after surgical treatment in patients with patellofemoral instability (PFI) and maltracking. We hypothesized that the direct effects of patella-realigning surgical procedures on dynamic patellar tracking can be visualized and verified radiologically. We further hypothesized that the common parameters of dynamic patellar tracking will improve after surgical correction.

## 2. Materials and Methods

### 2.1. Study Population

Patients who presented with a history of PFI and clinically lateral patellar maltracking between December 2019 and May 2022 were included in the study. The inclusion criteria were symptoms of PFI, such as recurring patella dislocations; clinically apparent patellar maltracking (increased mediolateral translation, positive [reversed] J sign); planned surgical realignment; and written consent to participate in the study (Table 1). Patients with a limited range of motion (ROM), acute joint effusion, concomitant knee injuries, or isolated patellar instability without signs of patellar maltracking were excluded from the study.

**Figure 1:**
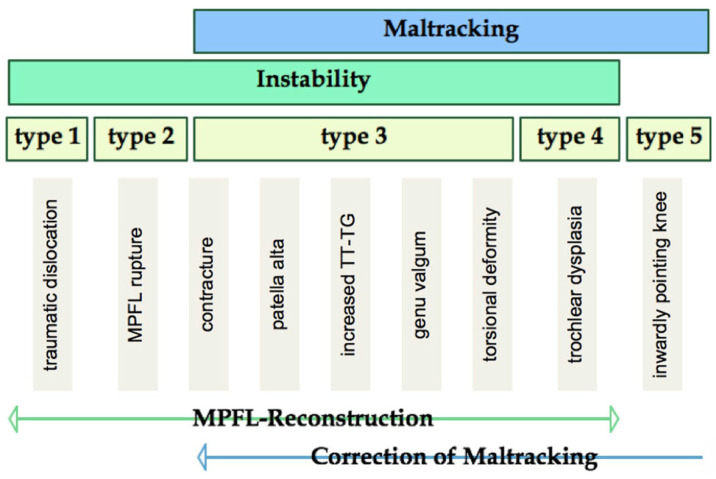
Algorithmic approach for the treatment of patellofemoral instabilities with or without patellar maltracking according to Frings et al. [26]. While isolated patellofemoral instabilities without patellar maltracking can be addressed by soft-tissue stabilizing techniques, a concomitant patellar maltracking requires additional diagnostic and therapeutic measures. Although closely connected, patellar instability and patellar maltracking should therefore be understood and treated as separate conditions. Adapted with permission from Ref. [26]. Copyright 2018 Springer Nature. Reprinted with permission.

**Figure 2:**
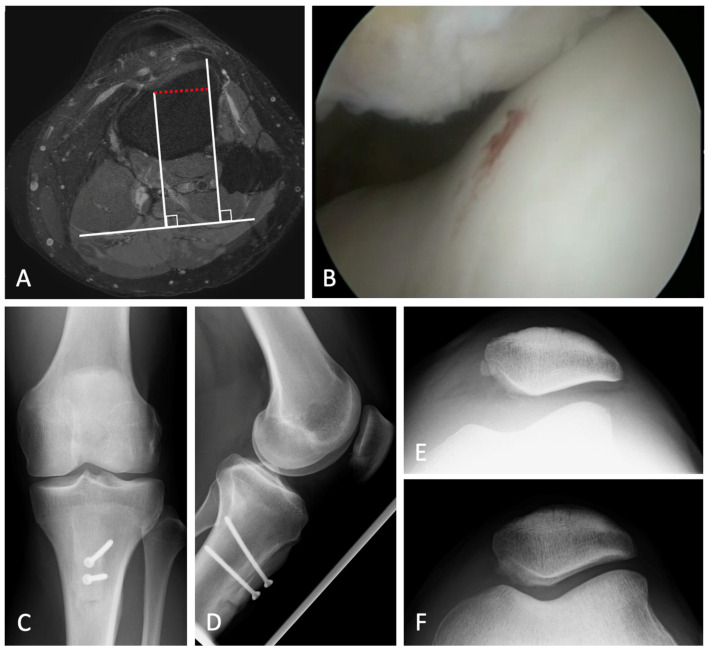
Lateral patellar maltracking in a left knee. Preoperative analysis revealed a TT-TG (tibial tubercle to trochlear groove) distance of 18 mm, with no further risk factors (**A**). Intraoperative arthroscopy (with low water pressure) confirmed a lateralized patellar tracking (**B**), reinforcing the indication to perform a medializing osteotomy of the tibial tubercle (**C**,**D**). A comparison of pre- and postoperative tangential radiographs of the patella showed a successful realignment of the patella (**E**,**F**).

Surgical realignment procedures were indicated and performed by three experienced orthopedic surgeons (MK, KF, and JF) on the basis of the clinical and radiological findings, following an algorithmic treatment approach (Figure 1 and Figure 2) [12,26]. The first radiological examination was performed prior to the planned surgery. The follow-up examination was performed at least 3 months postoperatively to ensure sufficient rehabilitation and an unrestricted ROM. The study design was approved by the local ethics committee (ID PV7101), and the study was conducted in accordance with the Good Clinical Practice Guidelines and the recommendations in the Declaration of Helsinki. Written consent was obtained for each patient or parent, if underage.

### 2.2. Surgical Techniques

**Figure 3:**
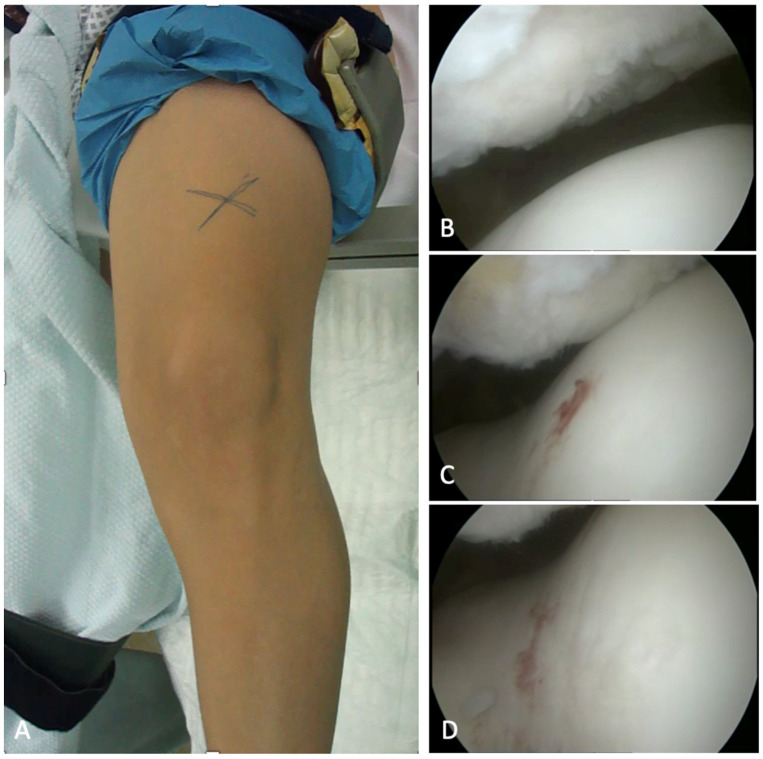
Patient set-up during surgery. The knees were placed in an electric leg holder with an inactive, but ready-to-use tourniquet (**A**). A diagnostic arthroscopy was performed at the beginning of surgery, in order to evaluate patellar tracking during passive range of motion, as well as the patellofemoral cartilage (**B**–**D**).

The knees were placed in an electric leg holder which comprised a ready-to-use tourniquet. Diagnostic arthroscopy was performed at the beginning of each procedure to allow for the assessment patellofemoral maltracking and to check for any concomitant (cartilaginous) injuries (Figure 3).

After arthroscopy, surgical correction of patellar maltracking was performed in accordance with the underlying pathology [12].

Lateralized tibial tubercles were addressed by either a medializing osteotomy in skeletally matured patients [14] or a soft tissue medialization of the patellar tendon insertion in skeletally immature patients [27]. In both cases, a 3 to 4 cm longitudinal skin incision was performed medial to the tibial tubercle to allow simultaneous harvesting of the gracilis tendon. For the osteotomy, a bony fragment of 5 cm in length was created with an oscillating saw, using the same skin incision. After longitudinal dissection of the distal lateral retinaculum, the tubercle fragment was medialized until a physiological TT-TG distance was achieved. It was then fixed with two K-wires, followed by osteosynthesis with two or three 3.5 mm bicortical screws. Soft tissue medialization was performed by sharp mobilization of the lateral periosteum, allowing the patella to shift medially during flexion [27]. The tendon insertion was then refixed to its new origin using two suture anchors (FastAk, Arthrex, Naples, FL, USA).

One valgus deformity was treated with a medial closed-wedge distal femoral osteotomy (DFO) [26]. After the malalignment test (MAT) and planning of the correction, a medial subvastus approach to the distal femur was developed. After careful preparation, the osteotomy and height of the medial wedge were marked with two guide wires. The osteotomy was performed with an oscillating saw. After removal of the wedge, the gap was closed and fixed using angle-stable plate osteosynthesis (Loqteq, aap Implants Inc., Berlin, Germany). The same surgical approach was used for one torsional DFO [16]. After the height of the osteotomy was marked using two guide wires, two Schanz pins marked the planned angle of the torsional correction. After the osteotomy was completed, rotation was performed until the Schanz pins were arranged in a parallel manner and fixed with a fixator clamp. Plate synthesis was performed as described earlier.

For trochlea dysplasia, sulcus-deepening trochleoplasty was performed in accordance with the technique described by Bereiter et al. [28]. Lateral arthrotomy was performed with lengthening of the lateral retinaculum [29]. An osteochondral flap was prepared using chisels, and excessive bone stock was carefully removed using PoweRasp (Arthrex, Naples, Italy). The flap was carefully molded to form a novel sulcus, and the osteotomy was fixed with a 3 mm Vicryl tape. In all cases, medial patellofemoral ligament (MPFL) reconstruction was performed with a doubled free gracilis tendon graft using a suture-anchor patellar fixation technique [30,31].

### 2.3. Examination Setup

For the radiological examination, a 3-Tesla MRI unit (Ingenia, Philips, Best, The Netherlands) was used in all subjects. The patient was placed in the supine position. First, a native MRI was performed using a standard 16-channel knee coil (Philips, Best, The Netherlands), with the knee placed in 20° flexion. The standard knee protocol consisted of fat-saturated PDw fast-spin echo (FSE) sequences in frontal, sagittal, and transverse plane orientations, and a T1-weighted (T1w) FSE sequence in a coronal orientation. For the acquisition of the FS Pdw sequence, the following parameters were chosen: repetition time (TR), 2387 ms; echo time (TE), 27 ms; flip angle, 90°; slice thickness, 1.6 mm; matrix, 704 × 704; field of view (FOV), 142 × 142 mm; number of slices, 180. The scan time for each knee was 11 min 47 s. The T1w FSE sequence was performed in coronal orientation (TR, 533 ms; TE, 8.7 ms; flip angle, 90°; slice thickness, 2.5 mm; matrix, 640 × 640; FOV, 180 × 180 mm; slices, 39; scan time, 2 min 24 s).

**Figure 4:**
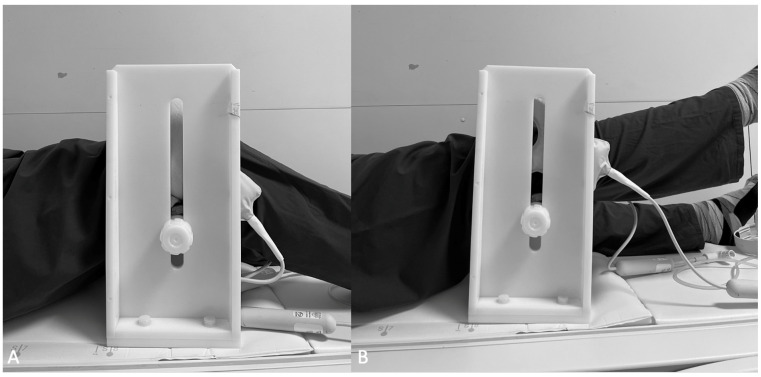
Examination set-up for dynamic magnetic resonance imaging (MRI). The patient’s knees were stored in 40° of flexion, using a customized positioning device (**A**). Two ring coils (Philip, Best, The Netherlands) were placed on either side of the knee. During image acquisition, the knees were actively moved between 40° and full extension (**B**).

**Figure 5:**
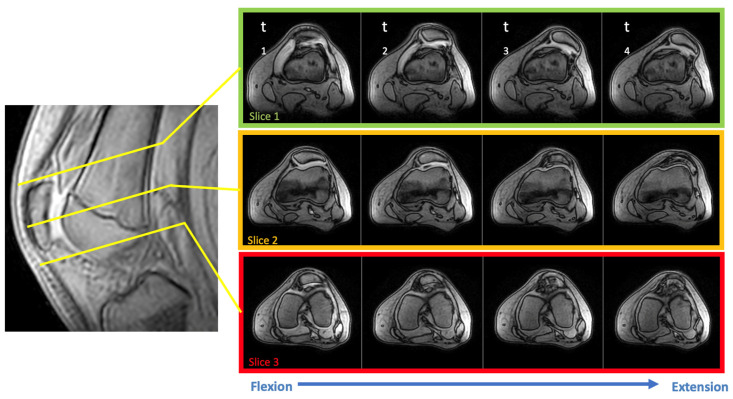
Real-time dynamic MRI measurement of patellar maltracking in the left knee of a symptomatic patient. In accordance with the technique described by Frings et al. [5], three slices in transverse orientation were followed by one sequence in sagittal plane orientation. Four measurement time points (t_1_–t_4_) are shown. During active repetition of knee flexion and extension, patellar tracking could be measured in real time.

Dynamic MR examination was performed in accordance with a previously described and validated dynamic MRI examination protocol [23,25]. The knees were placed in an individually adjustable custom positioning device, using two ring coils (Philips, Best, The Netherlands). In a resting position, the knee was kept in 40° flexion (Figure 4). Full extension of the knees within the gantry was ensured prior to the examination. A multi-slice spoiled gradient echo sequence was acquired with three slices in transverse orientation and one sequence in sagittal plane orientation. The levels of the three planes were referenced to the posterior femoral condyles, the deepest point of the trochlear groove, and the largest diameter of the patella in full extension. To allow acquisition of all relevant landmarks, the gap size was set to 12 mm, and a pulse angle of 8° was chosen for optimal contrast. The SENSE factor was 2; TR, 8.3 ms; TE, 1.32 ms; and temporal solution, 0.6 s [23]. Within a scanning time of 30 s, a total of 150 images were acquired. During this time frame, the patient was asked to perform two repetitive cycles of active extension and flexion, within the predefined ROM [23] (Figure 5).

## 3. Measurement and Analysis

MRI analysis was performed using Centricity RIS/PACS (GE, Boston, MA, USA).

Static MRI was used to examine the radiological parameters of anatomical PF risk factors. Patella height was determined using the Canton-Deschamps Index (CDI), with the knee placed in 20° flexion [32,33]. Furthermore, the distances between the tibial tubercule and the medial border of the posterior cruciate ligament (TT-PCL) [34] and the trochlear groove (TT-TG) [35] were measured. Trochlear geometry was assessed by measuring the trochlea sulcus angle, lateral trochlear inclination, and trochlear depth in the most proximal transversally oriented plane, showing the trochlea completely with cartilage [36,37,38,39].

**Figure 6:**
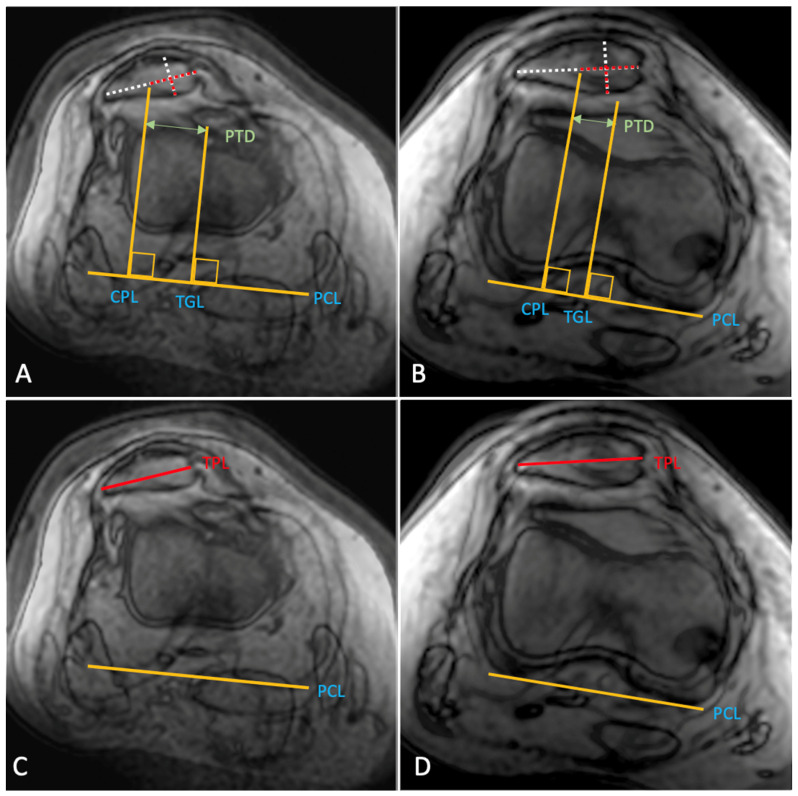
Preoperative and postoperative measurement of the proximal patella position in a fully extended right knee. Compared to the preoperative examination (**A**), the postoperative patellotrochlear distance (PTD) (**B**), measured between the deepest point of the trochlea groove (TGL) and the center-center position of the patella (CPL), was significantly reduced. Accordingly, the postoperative patellar tilt (angle between the transpatellar line [TPL] and the posterior condylar line [PCL]) was reduced significantly compared to the preoperative value (**C**,**D**). All measurements were performed on transversely oriented MRI slides, in accordance with the technique described by Frings et al. [23].

Dynamic mediolateral patella translation (dMPT) and dynamic patellar tilt (dPT) were used to define patellar tracking [23]. Both parameters were measured at two time points, before and at least 3 months after surgery, by an experienced orthopedic surgeon and a musculoskeletal radiologist (Figure 6). Results were referenced to the normal values according to Frings et al. [25].

### Statistical Calculations

All descriptive analyses were conducted using SPSS Statistics version 22.0.0 (SPSS, Inc., 2010, Chicago, IL, USA). Normal distribution was verified using the Kolmogorov-Smirnov test. Differences between the mean values of the kinematic parameters were calculated using the paired *t* test to calculate p values and Cohen’s effect size (*d*). A *p* value < 0.05 was considered statistically significant. Pearson’s correlation coefficient was calculated to determine whether the radiological parameters correlated with dMPT and dPT. Interrater reliability was tested by calculating the intraclass correlation coefficients (ICC) [40]. Correlation plots were built with Microsoft Excel version 16.47.1 (Microsoft, Redmont, WA, USA).

## 4. Results

Twenty-four patients (7 men and 17 women; mean age, 23.0 ± 8.5 years [range, 12–45 years]) who were surgically treated for patellar instability and maltracking between December 2019 and May 2022 were enrolled in the study. The (combined) surgical realignment procedures comprised bony tibial tubercle transfers (n = 10), soft tissue transfers of the patella tendon insertion (n = 5), trochleoplasty (n = 6), torsional distal femoral osteotomy (DFO; n = 1), lateral retinaculum lengthening (n = 3), and medially closing, varizating DFO (n = 1). All patients received an additional MPFL reconstruction for patellar stabilization. Table 2 presents the patient demographics and preoperative and postoperative presentations of the radiological parameters. 

**Figure 7:**
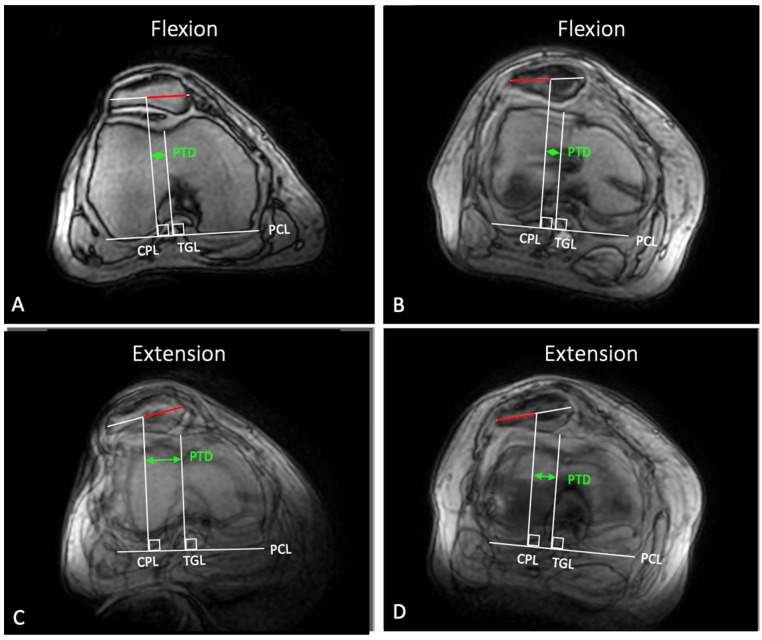
Correction of patellar maltracking in a right knee, caused by a femoral torsional deformity (−41° according to Waidelich et al. [41]). Preoperatively, the patellotrochlear distance (PTD) was 2.8 mm in 40° of flexion (**A**) and 16.6 mm in full extension (**C**), which resulted in a dynamic mediolateral patellar translation (dMPT) of 13.8 mm. Further, a dynamic patellar tilt (dPT) of 11° was found. After, torsional distal femoral osteotomy (DFO) and MPFL reconstruction, the PTD in flexion remained almost unchanged (**B**), but changed to 8 mm in full extension (**D**). Accordingly, a postoperative dMPT of 5 mm and a dPT of 6° was observed.

**Figure 8:**
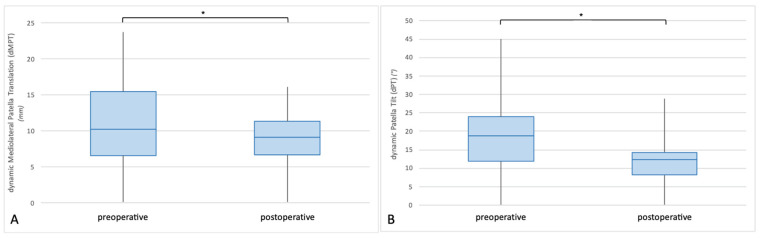
Boxplot illustration of preoperative vs. postoperative dynamic patellar tracking. After surgical realignment, both measurable parameters for dynamic patellar tracking had significantly improved, compared to the preoperative values. The dMPT had decreased from 10.95 ± 5.93 mm to 4.89 ± 0.40 mm (*p* < 0.001) (**A**). Accordingly, the dPT had changed from 14.50 ± 10.33° preoperatively to 8.44 ± 7.46° postoperatively (*p* = 0.026) (**B**).

The follow-up examination 3 months after surgery revealed significant changes of the dynamic parameters for patellar tracking (Figure 7). Dynamic mediolateral patellar translation (dMPT) was significantly reduced from 10.95 ± 5.93 mm preoperatively to 4.89 ± 0.40 mm postoperatively (*p* < 0.001, *d* = 1.410) (Figure 8), while the patella was positioned significantly more medially in extension (from 16.74 ± 6.32 mm to 10.83 ± 5.59 mm; *p* < 0.001, *d* = 0.989). Furthermore, a significant reduction in the dynamic patellar tilt (dPT), from 14.50° ± 10.33° to 8.44° ± 7.46°, was observed (*p* = 0.026, *d* = 0.668). In this regard, there were significant differences between the preoperative and postoperative patellar position and tilt in the fully extended knee.

Preoperative TT-TG showed a moderate correlation with preoperative dMPT (*p* = 0.013, *r* = 0.498) and a significant correlation with preoperative dPT (*p* = 0.012, *r* = 0.505). For both examiners (TD and JM), an excellent intra-rater correlation was found for the measurement of dMPT (ICC, 0.971; 95% confidence interval [CI], 0.941–0.983 and ICC, 0.973; 95% CI, 0.925–0.987, respectively) and dPT (ICC, 0.991; 95% CI, 0.980–0.994 and ICC, 0.992; 95% CI, 0.982–0.996, respectively). Between the two examiners, the inter-rater reliability was excellent for the measurement of presurgical dMPT (ICC, 0.977; 95% CI, 0.946–0.988) and dPT (ICC, 0.983; 95% CI, 0.960–0.993), and postsurgical dMPT (ICC, 0.955; 95% CI, 0.891–0.981) and dPT (ICC, 0.982; 95% CI, 0.956–0.992).

Clinically, all patients regained mobility and returned to an unrestricted level of activity in daily life. No recurring patellar dislocations were reported at the time of the postoperative follow-up examination.

## 5. Discussion and Conclusions

The most important finding of this study from using cinematic MRI was that surgical patellar realignment in patients with patellar instability and maltracking significantly improved the common dynamic parameters for patellofemoral tracking. For the first time, the postoperative success of patella-realigning procedures was visualized and verified radiologically in real patients.

With the development of algorithmic classification systems and a better understanding of the individual pathogeneses, surgical treatment of patellofemoral instabilities has become more precise [11,12]. Several clinical studies have outlined the effectiveness of procedures, such as trochleoplasty [42,43], torsional DFO [16,44], varizating DFO [26,45], or tibial tubercle transfer [14,46,47], in terms of redislocation rates, improvement of clinical scores, or return to activity. Clinical parameters and PROMs allow for a good estimation of the expected functional outcome. However, the true effect of an applied surgical procedure on patellofemoral tracking itself has been shown in biomechanical in vitro or cadaveric studies but remains invisible in clinical examination [7,22].

It is known that joint congruency, patellar tracking, contact pressure, and contact area are significantly altered in unstable patellofemoral joints [10,23,48]. This becomes particularly important on two occasions: cartilage deterioration due to persistent maltracking and revision surgery for failed patellar stabilization. Patellar maltracking is known to be associated with early cartilage deterioration in young patient populations [7,10,14,26,49]. Furthermore, despite the absence of recurring dislocations, occult persistence of maltracking can be held accountable for progressing joint degeneration in the long term [7,50,51,52]. Therefore, the goal of surgical therapy must be the best possible restoration of physiological patellofemoral kinematics, in addition to sufficient medial soft tissue stabilization [52].

Stephen et al. [7] analyzed the change in pressure distribution in the patellofemoral joint following tibial tubercle medialization in cadaveric knees. They found a significant reduction in lateral peak pressure, without adverse medial-sided effects [7]. In another biomechanical study, internal torsional deformities of the femur were related to an increased lateral patellar shift and tilt [6]. Another computational study identified trochlear dysplasia as a direct cause of lateral patellar maltracking [53]. Accordingly, trochleoplasty was found to decrease lateral patellar shift [54] and restore patellofemoral congruency [55]. Despite the consistent findings of these in vitro studies, they are only partially transferable to real patients [56,57,58]. In clinical practice, a measurable postoperative verification of restored patellar tracking could be beneficial and may help to predict and prevent patellofemoral osteoarthritis.

In the patient collective of the present study, both parameters for dynamic patellar tracking (dMPT and dPT) had significantly improved at follow-up 3 months after surgical correction. While the dPT was reduced by 42%, dMPT was found to be 45% of its preoperative distance (Figure 8). Correspondingly, the static radiological parameters for PFI and PM were corrected to physiological values. On closer inspection, it became apparent that the postoperative changes of dynamic patellar tracking were mainly caused by the significant changes in patellar position in extension, while the patellar position in flexion barely changed. Accordingly, surgical realignment was found to reduce the extent of proximal lateralization, thus correcting proximal patellar maltracking.

Nevertheless, compared with the normal values observed in healthy individuals, both dynamic parameters remained slightly increased and stayed outside of the defined normal range [25]. One possible explanation for this observation could be the postoperative impairment of quadriceps muscle function. Especially in postoperative conditions, quadriceps muscle (QM) hypotrophy has been shown to persist up to 6 months after even minor arthroscopic procedures [59,60]. The vastus medialis obliquus has a relevant influence on patellar tracking, so further potential for improvement may be presumed depending on muscular rehabilitation [56,61,62,63]. Regarding this, considerable discrepancies have been found between measured patellar tracking with and without active QM contribution [23,64]. On the other hand, this is the first study to evaluate and quantify postoperative patellar tracking. Consequently, no benchmark values have been established to define postoperative success in terms of dynamic realignment, even in the presence of an asymptomatic clinical condition. Whether successfully restored patella tracking should match with the normal values observed in the healthy population and to which extent this might affect the PF cartilage in the long term remain unclear.

In failed patellofemoral stabilization, however, substantial deviations of postoperative patellar tracking in the mid-term follow-up should be indicative for revision surgery [65]. In addition to the assessment of potentially disregarded risk factors, an additional dynamic evaluation will refine failure analysis and contribute to improved postoperative expectations [18].

This study has some limitations. The number of included cases was comparably small and several surgical techniques for patella realignment were included. Therefore, no subgroup analyses were performed to differentiate between the individual effects of each technique. However, all patients presented with PFI and measurable maltracking, which was the primary subject of this study, regardless of the predominantly underlying pathology. Furthermore, the primary purpose of this study was to test for the feasibility of the applied examination technique. In addition, the follow-up period of 3 months was comparably short and thus did not allow researchers to perform a functional analysis or reliable assessment of PROMs. In consideration of the study objective to gain immediate insight and a prompt postoperative control of success, however, the additional assessment of functional outcome measures was irrelevant for the confirmation of the hypothesis. 

Future studies should attempt to include larger numbers of cases, thereby allowing for a reliable subgroup analysis. In this regard, the individual influences of different anatomic risk factors on patellar tracking and the correction potentials of according surgical procedures could be of interest. Furthermore, a longer follow-up period might help to understand the persisting differences between postoperative and “normal” patellar tracking, especially if combined with clinical PROMs. Subsequently, an increase of statistical power will help to define benchmark values for successful realignment of patellar tracking. With ongoing technological advances, a cinematic three-dimensional acquisition of patellar tracking could further improve the understanding of patellofemoral kinematics by allowing synchronized multi-plane measurements.

## 6. Conclusions

Surgical patellar realignment contributed to the significant improvement of patellofemoral kinematics, with an approximation to normal values. The postoperative application of dynamic MRI allowed for a quantification of the performed correction. 

This may in turn allow for a postoperative control of success and provide the basis for failure analysis in cases of failed patellofemoral stabilization.

## Figures and Tables

**Table 1:** Inclusion and exclusion criteria.

Inclusion Criteria	Exclusion Criteria
recurring patellar dislocations	limited range of motion
clinical signs for patellar maltracking	absence of patellar maltracking (absence of patellar maltracking)
planned surgical realignment	concomitant knee injuries
written consent to participate	acute joint effusion

**Table 2:** Mean descriptive data of the static and dynamic parameters for patellar tracking in the study population.

Parameter	Preoperative (n = 24)	Postoperative (n = 24)	*p*
Side			
Left knee	12		
Right knee	12		
Sex			
Male	7		
Female	17		
Age (range) Years	23.0 (12–45)		
TT-TG (±SD), mm	15.35 ± 5.18	10.17 ± 4.11	0.008
TT-PCL (±SD), mm	23.03 ± 4.50	16.30 ± 5.16	<0.001
Trochlear sulcus angle (±SD), degrees	143.80 ± 12.65	139.35 ± 8.80	0.678
Trochlear sulcus depth (±SD), mm	4.84 ± 1.49	5.29 ± 1.52	0.217
Lateral inclination angle (±SD), degrees	19.58 ± 7.08	19.41 ± 5.99	0.482
Canton Deschamps Index (±SD)	1.33 ± 0.20	1.29 ± 0.18	0.158
Patellar position in flexion (±SD), mm	5.75 ± 3.86	5.68 ± 4.20	0.661
Patellar position in extension (±SD), mm	16.74 ± 6.32	10.83 ± 5.59	<0.001
Dynamic mediolateral translation (±SD), mm	10.95 ± 5.93	4.89 ± 0.40	<0.001
Patellar tilt in flexion (±SD), degrees	8.65 ± 6.80	7.67 ± 4.19	0.719
Patellar tilt in extension (±SD), degrees	23.15 ± 11.03	16.11 ± 7.29	0.007
Dynamic patellar tilt (±SD), degrees	14.50 ± 10.33 to lateral	8.44 ± 7.46 to lateral	0.026

TT-TG: tibial tuberosity-trochlear groove distance, TT-PCL: tibial tuberosity-posterior cruciate ligament distance, SD: standard deviation.

## Data Availability

The data presented in this study are available on request from the corresponding author. The data are not publicly available owing to local guidelines for data storage.

## References

[1] Arendt E.A., Askenberger M., Agel J., Tompkins M.A. (2018). Risk of Redislocation After Primary Patellar Dislocation: A Clinical Prediction Model Based on Magnetic Resonance Imaging Variables. Am. J. Sports Med..

[2] Previtali D., Milev S.R., Pagliazzi G., Filardo G., Zaffagnini S., Candrian C. (2020). Recurrent Patellar Dislocations Without Untreated Predisposing Factors: Medial Patellofemoral Ligament Reconstruction Versus Other Medial Soft-Tissue Surgical Techniques-A Meta-analysis. Arthroscopy.

[3] Hevesi M., Heidenreich M.J., Camp C.L., Hewett T.E., Stuart M.J., Dahm D.L., Krych A.J. (2019). The Recurrent Instability of the Patella Score: A Statistically Based Model for Prediction of Long-Term Recurrence Risk After First-Time Dislocation. Arthroscopy.

[4] Balcarek P., Oberthur S., Hopfensitz S., Frosch S., Walde T.A., Wachowski M.M., Schuttrumpf J.P., Sturmer K.M. (2014). Which patellae are likely to redislocate?. Knee Surg. Sports Traumatol. Arthrosc..

[5] Frings J., Balcarek P., Tscholl P.M., Liebensteiner M., Dirisamer F., Koenen P. (2020). Conservative versus surgical treatment for primary patellar dislocation—A systematic review to guide risk stratification. Deutsch Arztebl. Int..

[6] Kaiser P., Schmoelz W., Schoettle P., Zwierzina M., Heinrichs C., Attal R. (2017). Increased internal femoral torsion can be regarded as a risk factor for patellar instability—A biomechanical study. Clin. Biomech..

[7] Stephen J.M., Lumpaopong P., Dodds A.L., Williams A., Amis A.A. (2015). The effect of tibial tuberosity medialization and lateralization on patellofemoral joint kinematics, contact mechanics, and stability. Am. J. Sports Med..

[8] Tanaka M.J., Elias J.J., Williams A.A., Carrino J.A., Cosgarea A.J. (2015). Correlation Between Changes in Tibial Tuberosity-Trochlear Groove Distance and Patellar Position During Active Knee Extension on Dynamic Kinematic Computed Tomographic Imaging. Arthroscopy.

[9] Tischer T., Geier A., Lenz R., Woernle C., Bader R. (2017). Impact of the patella height on the strain pattern of the medial patellofemoral ligament after reconstruction: A computer model-based study. Knee Surg. Sports Traumatol. Arthrosc..

[10] Van Haver A., De Roo K., De Beule M., Labey L., De Baets P., Dejour D., Claessens T., Verdonk P. (2015). The effect of trochlear dysplasia on patellofemoral biomechanics: A cadaveric study with simulated trochlear deformities. Am. J. Sports Med..

[11] Liebensteiner M., Keiler A., El Attal R., Balcarek P., Dirisamer F., Giesinger J., Seitlinger G., Nelitz M., Keshmiri A., Frings J. (2021). Conservative versus tailored surgical treatment in patients with first time lateral patella dislocation: A randomized-controlled trial. J. Orthop. Surg. Res..

[12] Frosch K.H., Schmeling A. (2016). A new classification system of patellar instability and patellar maltracking. Arch. Orthop. Trauma. Surg..

[13] Bartsch A., Lubberts B., Mumme M., Egloff C., Pagenstert G. (2018). Does patella alta lead to worse clinical outcome in patients who undergo isolated medial patellofemoral ligament reconstruction? A systematic review. Arch. Orthop. Trauma. Surg..

[14] Frings J., Krause M., Wohlmuth P., Akoto R., Frosch K.H. (2018). Influence of patient-related factors on clinical outcome of tibial tubercle transfer combined with medial patellofemoral ligament reconstruction. Knee.

[15] Laidlaw M.S., Feeley S.M., Ruland J.R., Diduch D.R. (2018). Sulcus-Deepening Trochleoplasty and Medial Patellofemoral Ligament Reconstruction for Recurrent Patellar Instability. Arthrosc. Tech..

[16] Frings J., Krause M., Akoto R., Frosch K.H. (2019). Clinical Results after Combined Distal Femoral Osteotomy in Patients with Patellar Maltracking and Recurrent Dislocations. J. Knee Surg..

[17] Tscholl P.M., Wanivenhaus F., Centmaier-Molnar V., Camenzind R.S., Fucentese S.F. (2020). Clinical and radiological results after one hundred fifteen MPFL reconstructions with or without tibial tubercle transfer in patients with recurrent patellar dislocation-a mean follow-up of 5.4 years. Int. Orthop..

[18] Zimmermann F., Bortlein J., Milinkovic D.D., Balcarek P. (2020). Patient-Reported Outcomes After Revision Surgery for Failed Medial Patellofemoral Ligament Reconstruction: A Matched-Pair Analysis Including Correction of Predisposing Factors. Am. J. Sports Med..

[19] Gorbaty J.D., Varkey D.T., Hong I.S., Trofa D.P., Odum S.M., Piasecki D.P., Saltzman B.M., Fleischli J.E. (2021). Outcomes and reoperation rates after tibial tubercle transfer and medial patellofemoral ligament reconstruction: Higher revision stabilization in patients with trochlear dysplasia and patella alta. Knee Surg. Sports Traumatol. Arthrosc..

[20] Leite C.B.G., Santos T.P., Giglio P.N., Pecora J.R., Camanho G.L., Gobbi R.G. (2021). Tibial Tubercle Osteotomy With Distalization Is a Safe and Effective Procedure for Patients With Patella Alta and Patellar Instability. Orthop. J. Sports Med..

[21] Tan S.H.S., Ngiam E.H.K., Lim J.Y., Lim A.K.S., Hui J.H. (2021). Surgical Management of Patella Alta in Patellofemoral Instability: A Systematic Review and Meta-analysis. Orthop. J. Sports Med..

[22] Hiemstra L.A., O’Brien C.L., Lafave M.R., Kerslake S. (2021). Common Physical Examination Tests for Patellofemoral Instability Demonstrate Weak Inter-Rater Reliability. Arthrosc. Sports Med. Rehabil..

[23] Frings J., Dust T., Krause M., Ohlmeier M., Frosch K.H., Adam G., Warncke M., Maas K.J., Henes F.O. (2020). Objective assessment of patellar maltracking with 3 T dynamic magnetic resonance imaging: Feasibility of a robust and reliable measuring technique. Sci. Rep..

[24] Burke C.J., Kaplan D., Block T., Chang G., Jazrawi L., Campbell K., Alaia M. (2018). Clinical Utility of Continuous Radial Magnetic Resonance Imaging Acquisition at 3 T in Real-time Patellofemoral Kinematic Assessment: A Feasibility Study. Arthroscopy.

[25] Frings J., Dust T., Krause M., Frosch K.H., Adam G., Warncke M., Welsch G., Henes F.O., Maas K.J. (2022). Dynamic Mediolateral Patellar Translation Is a Sex- and Size-Independent Parameter of Adult Proximal Patellar Tracking Using Dynamic 3 Tesla Magnetic Resonance Imaging. Arthroscopy.

[26] Frings J., Krause M., Akoto R., Wohlmuth P., Frosch K.H. (2018). Combined distal femoral osteotomy (DFO) in genu valgum leads to reliable patellar stabilization and an improvement in knee function. Knee Surg. Sports Traumatol. Arthrosc..

[27] Kraus T., Lidder S., Svehlik M., Rippel K., Schneider F., Eberl R., Linhart W. (2012). Patella re-alignment in children with a modified Grammont technique. Acta Orthop..

[28] Bereiter H., Gautier E. (1994). Die Trochleaplastik als chirurgische Therapie der rezidivierenden Patellaluxation bei Trochleadysplasie des Femurs. Arthroskopie.

[29] Biedert R.M. (2010). Laterale Retinakulumverlängerung bei arthroskopischen Eingriffen. Arthroskopie.

[30] Ren B., Zhang X., Zhang L., Zhang M., Liu Y., Tian B., Zhang B., Zheng J. (2019). Isolated trochleoplasty for recurrent patellar dislocation has lower outcome and higher residual instability compared with combined MPFL and trochleoplasty: A systematic review. Arch. Orthop. Trauma Surg..

[31] Schottle P.B., Romero J., Schmeling A., Weiler A. (2008). anatomical reconstruction of the medial patellofemoral ligament using a free gracilis autograft. Arch. Orthop. Trauma. Surg..

[32] Caton J., Deschamps G., Chambat P., Lerat J.L., Dejour H. (1982). Patella infera. Apropos of 128 cases. Rev. Chir. Orthop. Reparatrice Appar. Mot..

[33] Yue R.A., Arendt E.A., Tompkins M.A. (2017). Patellar Height Measurements on Radiograph and Magnetic Resonance Imaging in Patellar Instability and Control Patients. J. Knee Surg..

[34] Seitlinger G., Scheurecker G., Högler R., Labey L., Innocenti B., Hofmann S. (2012). Tibial tubercle-posterior cruciate ligament distance: A new measurement to define the position of the tibial tubercle in patients with patellar dislocation. Am. J. Sports Med..

[35] Schoettle P.B., Zanetti M., Seifert B., Pfirrmann C.W., Fucentese S.F., Romero J. (2006). The tibial tuberosity-trochlear groove distance; a comparative study between CT and MRI scanning. Knee.

[36] Biedert R.M., Bachmann M. (2009). Anterior-posterior trochlear measurements of normal and dysplastic trochlea by axial magnetic resonance imaging. Knee Surg. Sports Traumatol. Arthrosc..

[37] Nacey N.C., Fox M.G., Luce B.N., Boatman D.M., Diduch D.R. (2020). Assessing Femoral Trochlear Morphologic Features on Cross-Sectional Imaging Before Trochleoplasty: Dejour Classification Versus Quantitative Measurement. AJR Am. J. Roentgenol..

[38] Paiva M., Blønd L., Hölmich P., Steensen R.N., Diederichs G., Feller J.A., Barfod K.W. (2018). Quality assessment of radiological measurements of trochlear dysplasia; a literature review. Knee Surg. Sports Traumatol. Arthrosc..

[39] Stefanik J.J., Zumwalt A.C., Segal N.A., Lynch J.A., Powers C.M. (2013). Association between measures of patella height, morphologic features of the trochlea, and patellofemoral joint alignment: The MOST study. Clin. Orthop. Relat. Res..

[40] Koo T.K., Li M.Y. (2016). A Guideline of Selecting and Reporting Intraclass Correlation Coefficients for Reliability Research. J. Chiropr. Med..

[41] Waidelich H., Strecker W., Schneider E. (1992). Computed tomographic torsion-angle and length measurement of the lower extremity. The methods, normal values and radiation load. RoFo.

[42] Hiemstra L.A., Peterson D., Youssef M., Soliman J., Banfield L., Ayeni O.R. (2019). Trochleoplasty provides good clinical outcomes and an acceptable complication profile in both short and long-term follow-up. Knee Surg. Sports Traumatol. Arthrosc..

[43] Zaffagnini S., Previtali D., Tamborini S., Pagliazzi G., Filardo G., Candrian C. (2019). Recurrent patellar dislocations: Trochleoplasty improves the results of medial patellofemoral ligament surgery only in severe trochlear dysplasia. Knee Surg. Sports Traumatol. Arthrosc..

[44] Dickschas J., Tassika A., Lutter C., Harrer J., Strecker W. (2017). Torsional osteotomies of the tibia in patellofemoral dysbalance. Arch. Orthop. Trauma. Surg..

[45] Dickschas J., Ferner F., Lutter C., Gelse K., Harrer J., Strecker W. (2018). Patellofemoral dysbalance and genua valga: Outcome after femoral varisation osteotomies. Arch. Orthop. Trauma. Surg..

[46] Mulliez A., Lambrecht D., Verbruggen D., Van Der Straeten C., Verdonk P., Victor J. (2015). Clinical outcome in MPFL reconstruction with and without tuberositas transposition. Knee Surg. Sports Traumatol. Arthrosc..

[47] Chen H., Zhao D., Xie J., Duan Q., Zhang J., Wu Z., Jiang J. (2017). The outcomes of the modified Fulkerson osteotomy procedure to treat habitual patellar dislocation associated with high-grade trochlear dysplasia. BMC Musculoskelet. Disord..

[48] Clark D., Stevens J.M., Tortonese D., Whitehouse M.R., Simpson D., Eldridge J. (2019). Mapping the contact area of the patellofemoral joint: The relationship between stability and joint congruence. Bone Jt. J..

[49] Vollnberg B., Koehlitz T., Jung T., Scheffler S., Hoburg A., Khandker D., Hamm B., Wiener E., Diederichs G. (2012). Prevalence of cartilage lesions and early osteoarthritis in patients with patellar dislocation. Europ. Radiol..

[50] Salonen E.E., Magga T., Sillanpaa P.J., Kiekara T., Maenpaa H., Mattila V.M. (2017). Traumatic Patellar Dislocation and Cartilage Injury: A Follow-up Study of Long-Term Cartilage Deterioration. Am. J. Sports Med..

[51] Fick C.N., Grant C., Sheehan F.T. (2020). Patellofemoral Pain in Adolescents: Understanding Patellofemoral Morphology and Its Relationship to Maltracking. Am. J. Sports Med..

[52] Gobbi R.G., Demange M.K., de Avila L.F.R., Araujo Filho J.A.B., Moreno R.A., Gutierrez M.A., de Sa Rebelo M., Tirico L.E.P., Pecora J.R., Camanho G.L. (2017). Patellar tracking after isolated medial patellofemoral ligament reconstruction: Dynamic evaluation using computed tomography. Knee Surg. Sports Traumatol. Arthrosc..

[53] Rezvanifar S.C., Flesher B.L., Jones K.C., Elias J.J. (2019). Lateral patellar maltracking due to trochlear dysplasia: A computational study. Knee.

[54] Elias J.J., Rezvanifar S.C., Koh J.L. (2021). Groove-deepening trochleoplasty reduces lateral patellar maltracking and increases patellofemoral contact pressures: Dynamic simulation. J. Orthop. Res..

[55] Balcarek P., Zimmermann F. (2019). Deepening trochleoplasty and medial patellofemoral ligament reconstruction normalize patellotrochlear congruence in severe trochlear dysplasia. Bone Jt. J..

[56] Lorenz A., Muller O., Kohler P., Wunschel M., Wulker N., Leichtle U.G. (2012). The influence of asymmetric quadriceps loading on patellar tracking--an in vitro study. Knee.

[57] Stephen J., Alva A., Lumpaopong P., Williams A., Amis A.A. (2018). A cadaveric model to evaluate the effect of unloading the medial quadriceps on patellar tracking and patellofemoral joint pressure and stability. J. Exp. Orthop..

[58] Thomeer L., Guan S., Gray H., Schache A., de Steiger R., Pandy M. (2021). Six-Degree-of-Freedom Tibiofemoral and Patellofemoral Joint Motion During Activities of Daily Living. Ann. Biomed. Eng..

[59] Sherman S.L., DiPaolo Z.J., Ray T.E., Sachs B.M., Oladeji L.O. (2020). Meniscus Injuries: A Review of Rehabilitation and Return to Play. Clin. Sports Med..

[60] Hauger A.V., Reiman M.P., Bjordal J.M., Sheets C., Ledbetter L., Goode A.P. (2018). Neuromuscular electrical stimulation is effective in strengthening the quadriceps muscle after anterior cruciate ligament surgery. Knee Surg. Sports Traumatol. Arthrosc..

[61] Balcarek P., Oberthur S., Frosch S., Schuttrumpf J.P., Sturmer K.M. (2014). Vastus medialis obliquus muscle morphology in primary and recurrent lateral patellar instability. BioMed Res. Int..

[62] Pal S., Besier T.F., Draper C.E., Fredericson M., Gold G.E., Beaupre G.S., Delp S.L. (2012). Patellar tilt correlates with vastus lateralis: Vastus medialis activation ratio in maltracking patellofemoral pain patients. J. Orthop. Res..

[63] Sawatsky A., Bourne D., Horisberger M., Jinha A., Herzog W. (2012). Changes in patellofemoral joint contact pressures caused by vastus medialis muscle weakness. Clin. Biomech..

[64] Amis A.A., Senavongse W., Bull A.M. (2006). Patellofemoral kinematics during knee flexion-extension: An in vitro study. J. Orthop. Res..

[65] Kita K., Tanaka Y., Toritsuka Y., Amano H., Uchida R., Takao R., Horibe S. (2015). Factors Affecting the Outcomes of Double-Bundle Medial Patellofemoral Ligament Reconstruction for Recurrent Patellar Dislocations Evaluated by Multivariate Analysis. Am. J. Sports Med..

